# A Time-Based and Intratumoral Proteomic Assessment of a Recurrent Glioblastoma Multiforme

**DOI:** 10.3389/fonc.2016.00183

**Published:** 2016-08-22

**Authors:** Priscila F. de Aquino, Paulo Costa Carvalho, Fábio C. S. Nogueira, Clovis Orlando da Fonseca, Júlio Cesar Thomé de Souza Silva, Maria da Gloria da Costa Carvalho, Gilberto B. Domont, Nilson I. T. Zanchin, Juliana de Saldanha da Gama Fischer

**Affiliations:** ^1^Laboratory of Microbial Diversity from Amazon with Importance for Health, Instituto Leônidas e Maria Deane, Fiocruz, Manaus, Amazonas, Brazil; ^2^Laboratory for Proteomics and Protein Engineering, Carlos Chagas Institute, Fiocruz, Curitiba, Paraná, Brazil; ^3^Laboratory of Toxinology, Instituto Oswaldo Cruz, Fiocruz, Rio de Janeiro, Brazil; ^4^Laboratory for Protein Chemistry, Chemistry Institute, Federal University of Rio de Janeiro, Rio de Janeiro, Brazil; ^5^Department of General and Specialized Surgery, Antonio Pedro University Hospital, Fluminense Federal University, Rio de Janeiro, Brazil; ^6^Department of Neurosurgery, Ipanema Federal Hospital, Rio de Janeiro, Brazil; ^7^Laboratory of Molecular Pathology, Department of Pathology, University Hospital Clementino Fraga Filho, Federal University of Rio de Janeiro, Rio de Janeiro, Brazil

**Keywords:** glioblastoma multiforme, molecular heterogeneity, quantitative proteomics, iTRAQ

## Abstract

Tumors consist of cells in different stages of transformation with molecular and cellular heterogeneity. By far, heterogeneity is the hallmark of glioblastoma multiforme (GBM), the most malignant and aggressive type of glioma. Most proteomic studies aim in comparing tumors from different patients, but here we dive into exploring the intratumoral proteome diversity of a single GBM. For this, we profiled tumor fragments from the profound region of the same patient’s GBM but obtained from two surgeries a year’s time apart. Our analysis also included GBM‘s fragments from different anatomical regions. Our quantitative proteomic strategy employed 4-plex iTRAQ peptide labeling followed by a four-step strong cation chromatographic separation; each fraction was then analyzed by reversed-phase nano-chromatography coupled on-line with an Orbitrap-Velos mass spectrometer. Unsupervised clustering grouped the proteomic profiles into four major distinct groups and showed that most changes were related to the tumor’s anatomical region. Nevertheless, we report differentially abundant proteins from GBM’s fragments of the same region but obtained 1 year apart. We discuss several key proteins (e.g., S100A9) and enriched pathways linked with GBM such as the Ras pathway, RHO GTPases activate PKNs, and those related to apoptosis, to name a few. As far as we know, this is the only report that compares GBM fragments proteomic profiles from the same patient. Ultimately, our results fuel the forefront of scientific discussion on the importance in exploring the richness of subproteomes within a single tissue sample for a better understanding of the disease, as each tumor is unique.

## Introduction

Glioblastoma multiforme (GBM) is by far the most malignant and aggressive type of glioma. Despite the conventional treatments such as surgical resection, radiotherapy, and chemotherapy, GBM is likely to recur. The approximate median survival rate for recurrent GBM patients after surgery is of 7.4 months (1), so to comprehend this disease, an understanding of the molecular mechanisms inherent from recurrence is fundamental (2). Furthermore, due to heterogeneity, glioblastomas, and gliomas in general, do not present well-defined surgical margins (3).

In a previous report, transcriptomic data revealed that, for most cases, fragments from the same GBM sample could be classified into at least two molecular subgroups (4); needless to say, tumors consist of cells in different stages of transformation, resulting in both molecular and cellular heterogeneity. We recently assessed the proteomic profile of two different morphological regions from the same GBM sample derived from a formalin-fixed paraffin-embedded tissue (FFPE) (5) and found proteins exclusively identified in each region. These results corroborate on the postulation of the great variability, within the a single GBM, be it at the transcriptomic or proteomic level (6). Aquino et al. used a Multidimensional Protein Identification Technology (MudPIT) (7) to compare the protein profile present in gastric cancer biopsies against its respective resection margin and healthy tissue acquired from endoscopic patients (8). The authors suggested that the resection margin “seemed more proteomically alike” to cancer than the healthy tissue (8). In another work from Aquino et al., a gastric cancer biopsy was sectioned into ten parts and then each part was analyzed by MudPIT. The authors pinpointed tissue-specific proteins along the entire stomach with different levels of abundancy (9).

The aforementioned studies reflect difficulties and challenges in the inter and intratumoral assessment. In fact, large-scale intratumor heterogeneity assessments have been tremendously overlooked, most possibly because of technical difficulties. Nevertheless, the study of intratumor heterogeneity is now recognized as one of the main areas in the study of cancer, culminating in ever-increasing evidence that intratumor heterogeneity is the key to understanding treatment failure (10). The reasons are many, heterogeneity affects the tumor biology in various ways; for example, through the generation of chemoresistant microenvironment, or even rendering mutations that could be linked with a therapy failure (3). The understanding of such heterogeneity is fundamental for designing treatments that are more effective.

Here, we perform a GBM intratumoral heterogeneity study by comparing tumor fragments from the same patient, but obtained from surgeries held 1 year apart. Our quantitative proteomic assessment was performed by labeling the tryptic peptides with Isobaric Tags for Relative and Absolute Quantitation (iTRAQ) (11) and then performing a four-step offline MudPIT with an Orbitrap Velos mass spectrometer. Our study includes proteomic profiling of the profound, intermediary, and external regions of the GBM tumor.

## Case Presentation

The University Hospital Ethics committee approved this study under the following permit: CONEP 9681 no. 124 25000.009267/2004-25. A written informed consent was acquired from the patient.

**Figure 1:**
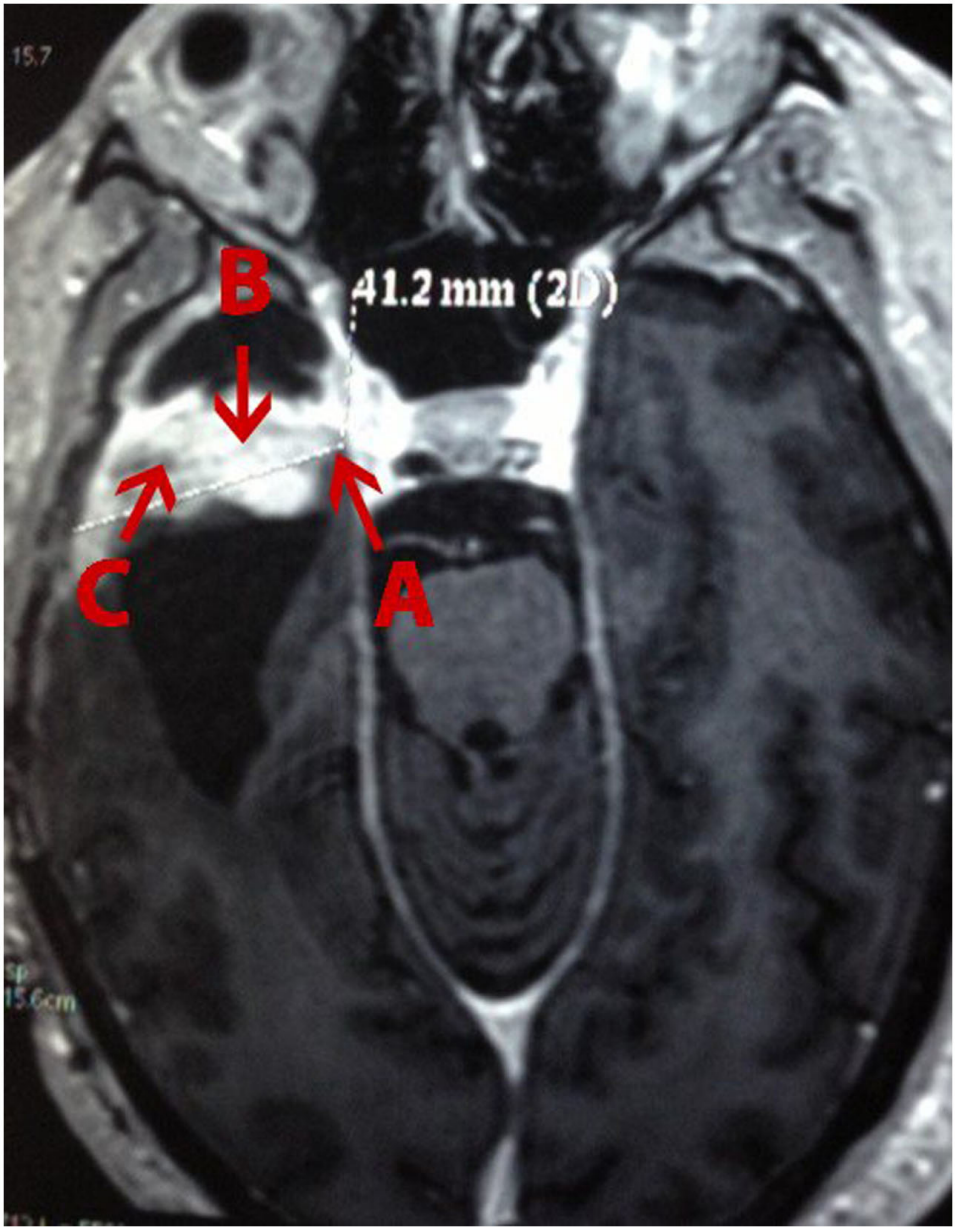
**Magnetic resonance imaging (MRI) of a patient with recurrent glioblastoma multiforme (GBM) in the right temporal lobe**. **(A)**, external; **(B)**, profound; and **(C)**, intermediary (as in according to the tumor’s center) area of the GBM.

A 49-year-old white man presenting painful headaches, drowsiness, and dysarthria was admitted to the neurosurgery service. A brain magnetic resonance imaging (MRI) scan revealed a regular space-occupying lesion in the right temporal lobe that was enhanced with gadolinium. MRI revealed presence of a right temporal mass with an intense surrounding vasogenic edema. The patient promptly (December, 2011) underwent a right temporal craniotomy with total tumor resection that was also confirmed by MRI. In order to control the intracranial hypertension, the patient was treated with dexametazona before and after surgery. Histological analysis carried out by three different pathologists confirmed the diagnosis of a GBM. The patient was treated with hydantoin and subjected to radiotherapy concomitantly with chemotherapy using temozolomide. After 15 days, the patient was accepted to join the phase I/II protocol of intranasal administration of 55 mg of the monoterpene perillyl alcohol four times a day (12–14). One year later, the patient returned to the same hospital presenting similar symptoms and another MRI was obtained (Figure 1). The patient underwent a second neurosurgery due to the recurrence of the GBM.

One tumor fragment from the profound region was obtained during the first surgery and three tumor fragments, from different regions (i.e., profound, external, and intermediary) (Figure 1), from the second surgery. All fragments were stored at −80°C for further proteomic analysis.

## Experimental Section

### Materials

Isobaric Tags for Relative and Absolute Quantitation (Cat. no 4352135) and Self-Pack Poros 20 R2 resin (Cat. no 1112810) were acquired from Applied Biosystems. Qubit^®^ Protein Assay Kit (Cat. no Q33212) and RapiGest SF acid-labile surfactant (Cat. no 186001861) were purchased from Invitrogen (Carlsbad, CA, USA) and Waters Corp. (Milford, MA, USA), respectively. Sequence grade modified trypsin (V511A) was purchased from Promega. Strong Cation Exchange Macro Spin Column (Cat. no 744202) was acquired from Harvard Apparatus. All other laboratory reagents were acquired from Sigma-Aldrich (St. Louis, MO, USA), unless specified otherwise.

### Sample Preparation and Protein Digestion

All four glioblastoma sample lysates were obtained by sonication in ice, with amplitude of 30% for three cycles of one and a half minute each (Hielscher – UP50H) immerse in a solution containing 0.2% of RapiGest™ (w/v) in 50 mM triethylammonium bicarbonate. Then, the lysates were centrifuged at 20,000 × *g* during 30 min at 4°C. Subsequently, the protein content was quantified by a fluorimetric assay using the Qubit^®^ 2.0 platform according to the manufacturer’s instructions. Two hundred micrograms of protein from each region were reduced with 20 mM of Tris (2-carboxyethyl) phosphine (TCEP) at 60°C for 30 min. Afterward, all samples were cooled to room temperature and incubated in the dark with 66 mM of iodoacetamide (IAA) for 30 min.

Afterward, the samples were digested for 20 h with sequence grade modified trypsin (Promega) at a 1:50 (E/S) ratio at 37°C. Following digestion, all reactions were acidified with 10% (v/v) trifluoroacetic acid (0.5% v/v final concentration) to stop proteolysis and degrade RapiGest. The samples were centrifuged for 30 min at 20,000 × *g* at 20°C to remove insoluble materials.

### Isobaric Tags for Relative and Absolute Quantitation Labeling

The peptides were desalted with Poros^®^ R2 resin (*Applied Biosystems*) packed “in house” according, to the manufacturer’s instructions. Twenty micrograms of tryptic peptides obtained from each area of the glioblastoma (i.e., profound from the first surgery, profound, intermediary, and external area from the second surgery) were labeled with the iTRAQ reagents according to the manufacturer’s directions. Briefly, for each iTRAQ 4-plex reagent vial, 70 μL of ethanol was individually added at room temperature. Each vial was vortexed and its contents were transferred to another tube containing twenty micrograms of digested peptides and labeled as follows: profound area from first surgery with iTRAQ-114, and profound, intermediary, and external areas from the second surgery labeled with iTRAQ-115 (Figure 1), iTRAQ-116 (Figure 1), and iTRAQ-117 (Figure 1), respectively. Subsequently, the samples were incubated for 1 h at room temperature. Labeled peptides were combined in a single tube and concentrated on a SpeedVac (Thermo Fischer Scientific).

### Strong Cation Exchange Chromatograph

The labeled peptides were suspended in solution A (5 mM KH_2_PO_4_ + 25% ACN, pH 3) and loaded into a strong cation exchange macro spin column from Harvard Apparatus. Peptides were eluted from the column in a stepwise manner by applying solution A with increasing KCl concentrations of 75, 150, 250, and 350 mM. Each fraction was desalted once again with Poros R2 resin 20 μm (Applied Biosystems) according, to manufacturer’s instructions. The flow-through was also desalted and stored for posterior analysis.

### Mass Spectrometry Analysis

The desalted peptide mixture was analyzed three times as follows. The setup used an *Easy*-nLC II (Proxeon) coupled online with a LTQ-Orbitrap Velos (Thermo, San Jose, CA) mass spectrometer (15). The peptide mixtures were loaded in a pre-column (2 cm, 200 μm inner diameter, 5 μm C18 beads Reprosil-AQ Pur, Dr. Maisch) and were chromatographically separated using a 20 cm analytical column (75 μm inner diameter) that was packed in-house with 3 μm C18 beads (Reprosil-AQ Pur, Dr. Maisch). The flow rate was 200 nL/min and the mobile phase composition was 5% acetonitrile in 0.1% formic acid. We then applied a 120 min gradient using first 5–40% acetonitrile in 0.1% formic acid for 100 min, then 40−95% acetonitrile in 0.1% formic acid for 20 min. The effluent from the nLC column was directly electrosprayed into the mass spectrometer.

The LTQ-Orbitrap Velos instrument was set in data dependent mode to automatically switch between full scan MS and MS/MS acquisition with a dynamic exclusion of 20 s turned on. Survey scans (350–2,000 *m/z*) were acquired in the Orbitrap system with a resolution of 60,000 at *m/z* 110. The ten most intense ions with charge states of 2+ or 3+ were sequentially isolated and fragmented in the HCD collision cell using a normalized collision energy of 40. The fragment ions were analyzed with a resolution of 7,500. The general mass spectrometric conditions were as follows: 2.30 kV spray voltage, 100 μA source current, no sheath and auxiliary gas flow, heated capillary temperature of 225°C, predictive automatic gain control (AGC) enabled, and an S-lens RF level of 64%. Mass spectrometer scan functions and nLC solvent gradients were controlled by the Xcalibur data system (Thermo, San Jose, CA, USA).

### Peptide Spectrum Matching

Sequences from *Homo sapiens* were downloaded from the UniProt consortium. A target-decoy database was generated using PatternLab 4.0 (16) to include a reversed version of each sequence found in the database plus those from 127 common mass spectrometry contaminants. The ProLuCID search engine (v. 1.3.1) (17) was used for comparing experimental spectra against those theoretically generated from a sequence database. The search was limited to fully and semi-tryptic peptide candidates. The search parameters imposed carbamidomethylation of cysteine as a fixed modification and the iTRAQ-4 modification in the N-terminal, K, and Y residues as variable. The search engine accepted peptide candidates within a 40-ppm tolerance from the measured precursor *m/z* and used the XCorr as the primary search engine score.

### Assessment of Peptide Sequence Matches and Profile Grouping

The Search Engine Processor (SEPro), built into PatternLab 4.0, was used for converging to a list of identifications with less than 1% of false discovery rate (FDR) at the protein level, as previously described (18). Briefly, the identifications were grouped by charge state (2+ and ≥3+), and then by tryptic status, resulting in four distinct subgroups. For each group, the XCorr, DeltaCN, DeltaPPM, and Peaks Matched values were used to generate a Bayesian discriminator. The identifications were sorted in non-decreasing order according to the discriminator score. A cutoff score was established to accept a false-discovery rate (FDR) of 1% at the peptide level based on the number of labeled decoys. This procedure was independently performed on each data subset, resulting in an FDR that was independent of charge state or tryptic status. Additionally, a minimum sequence length of six amino-acid residues was required. Results were post-processed to only accept peptide spectrum match (PSMs) with less than 6 ppm from the global identification average. One-hit wonders (i.e., proteins identified with only one mass spectrum) with the peptide having an XCorr of less than 2.5 were discarded. This last filter led to FDRs, now at the protein level, to be lower than 1% for all search results.

PatternLab’s TrendQuest module was used for grouping the proteomic profiles from each protein according the signals provided by the corresponding reporter ions as previously performed (19). PatternLab’s isobaric analyzer module was employed for pinpointing differentially abundant proteins when comparing the profound tissue samples from the first and second surgery as described in our bioinformatics protocol (16). PatternLab’s Gene Ontology Explorer module (20) and www.reactome.org (21) were used to help interpret the data.

## Results and Discussion

**Figure 2:**
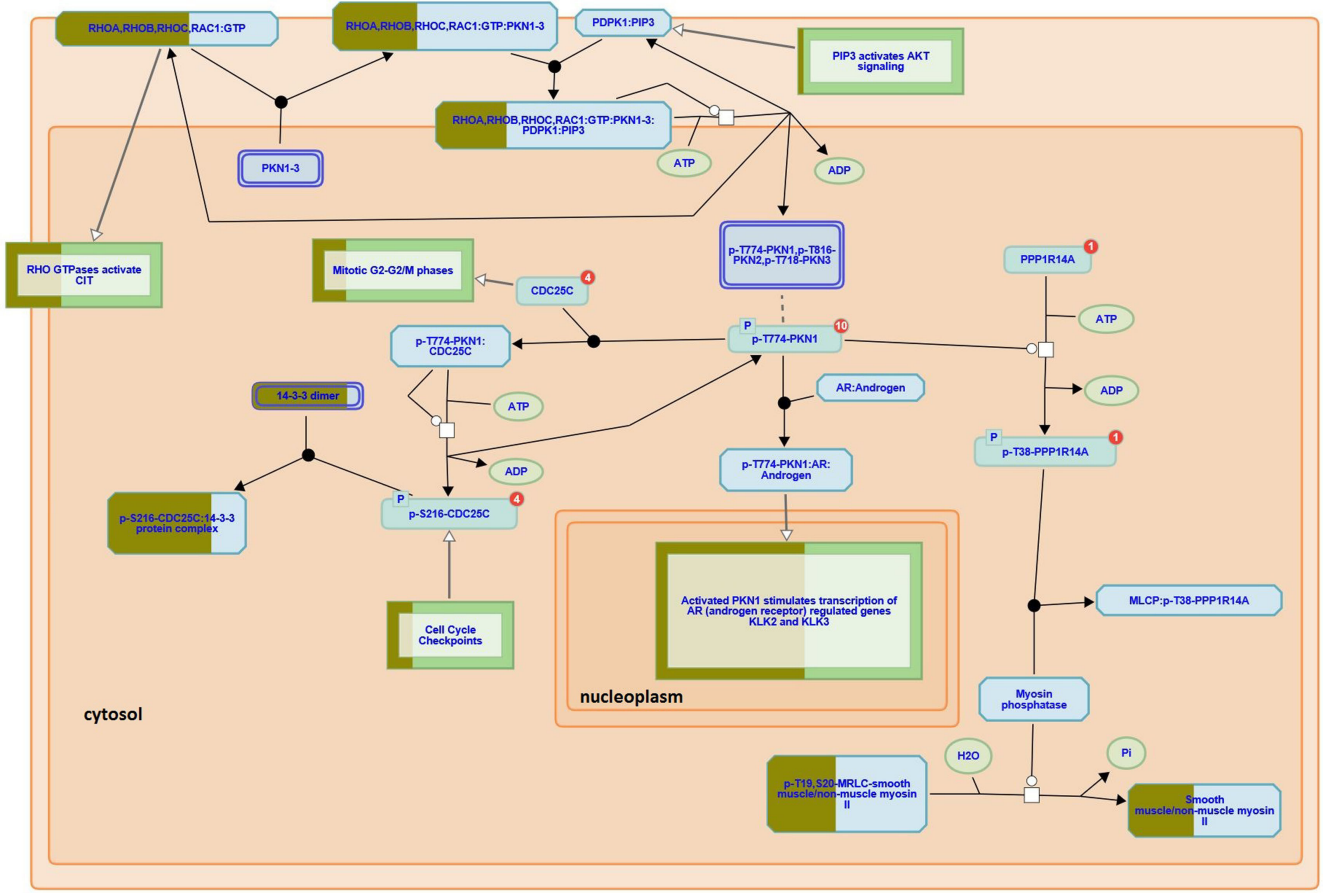
**RHO GTPases activate PKNs pathway (reactome identifier: R-HSA-5625740)**. The diagram represents a curated pathway provided from www.reactome.org generated with our data. The circles and boxes represent small molecules and sets of proteins, respectively. Some protein sets are partially filled with dark green representing the percentage of the proteins we identified in that collection.

Here we used iTRAQ to study the proteome diversity from three different areas of a recurrent GBM tumor i.e., profound, intermediary, and external, as described in the experimental section. We also compared proteomic profiles of tumor fragments from the same patient’s GBM profound area, but obtained from surgeries held a year apart. Our proteomic results identified 18,929 mass spectra providing sequences of 3,779 peptides (FDR = 0.29%) that map to 2,773 (FDR = 0.97%) protein sequences, of which 2,742 were successfully quantitated by iTRAQ; this list can be simplified to 768 proteins according to the bipartite-graph analysis for maximum parsimony (22). We employed the reactome software to obtain a birds-eye view of enriched pathways; for this, we provided as input all proteins quantitated by iTRAQ. The reactome pointed 21 enriched pathways (*p* < 0.01); a full list (enriched or not) is provided in Table S1 in Supplementary Material. Among the enriched pathway diagrams, we highlight the “RHO GTPases activate PKNs,” of which we identified 31 out of the 60 possible proteins from this pathway (Figure 2). PKNs, or protein kinase C-related kinases, feature a C-terminal serine/threonine kinase domain and three RHO-binding motifs at the N-terminus. PKNs play key roles in the regulation of cell cycle, receptor trafficking, vesicle transport, apoptosis, and mediating ligand-dependent transcriptional activation by the androgen receptor, to name a few. According to Fortin Ensign et al., in GBMs, Rho GTPases are usually deregulated, generally by hyperactivity or overexpression of their activators, Rho GEFs (Guanine Nucleotide Exchanging Factor); this ultimately promotes invasiveness and survival of the glioma cell (23).

**Figure 3:**
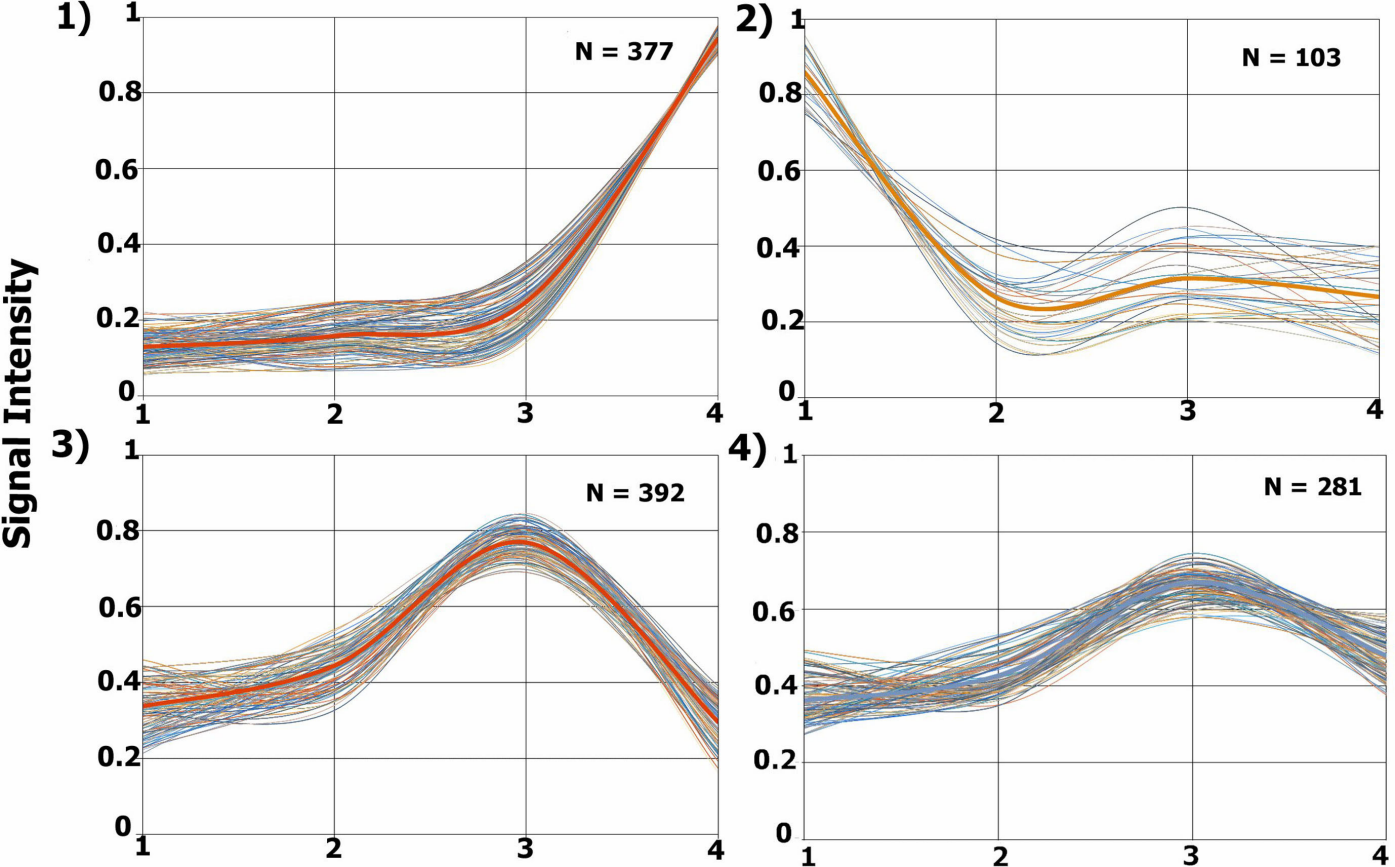
**Proteomic profile clusters**. Each panel displays the expression profiles of proteins that were grouped according to their abundance derived from each glioblastoma multiforme (GBM) condition. Each line represents a protein’s relative abundance. A thicker line is found in each cluster, this line is obtained by averaging the values at each point. The *y*-axis shows the normalized iTRAQ intensity. The *x*-axis represents the following conditions: 1, profound tissue area of a GBM from the first surgery (iTRAQ-114); 2, profound tissue area of a GBM from the second surgery (iTRAQ-115); 3, intermediary tissue area of a GBM from the second surgery (iTRAQ-116), and 4, external tissue area of a GBM from the second surgery (iTRAQ-117).

The TrendQuest module generated four major protein groups by clustering proteins according to their relative abundance in each of the four samples under consideration (Figure 3). We note that not all identifications belong into one of these four groups. The clusters 1, 2, 3, 4 are composed of 377, 103, 392, and 281 protein entries, respectively. A visual assessment in Figure 3 reveals that Cluster #1 relates to proteins with higher abundancy in the external tissue (second surgery), #2 high in the profound tissue (first surgery), #3 high in intermediary tissue (second surgery), and #4, high in the intermediary (second surgery) and slightly less in the external tissue (second surgery). TrendQuest’s unsupervised clustering provides a bird’s-eye view of the proteomic landscape throughout the four biological conditions. Figure 3 reveals that more changes occurred when comparing the different anatomical regions of the same tumor (i.e., surgery 2) versus the comparison of the profound region of surgery 1 (point 1) versus that of surgery 2 (point 2). This conclusion emerges as cluster 2, linking to differentially abundant proteins between profound from surgery 1 and 2, is only accountable for 103 proteins; the remaining clusters (1050 proteins), show major changes among the different anatomical regions from surgery 2 and, arguably, no changes between surgery 1 and 2.

The list of the proteins from each cluster, together with their summed reporter ion signal, is provided in the Tables S2–S5 in Supplementary Material, respectively. Table S6 in Supplementary Material lists 27 proteins belonging to a (small) cluster that presented the least changes as in according to our visual inspection; we believe this cluster indirectly reflect the heterogeneity of the tumor. Quantitated proteins not belonging to one of these clusters are listed in Table S7 in Supplementary Material. A complete list of all identified proteins with their corresponding peptide identifications is available in Table S8 in Supplementary Material. We make all our raw mass spectrometry files, SEPro results and intermediary ProLuCID/PatternLab files available for downloading at http://proteomics.fiocruz.br/supplementaryfiles/oncology2016

Several reports corroborate with our results at the genomic and transcriptomic levels. Sottoriva et al. examined intratumoral heterogeneity of the external and profound areas of eleven GBMs at genomic and transcriptomic levels and concluded that fragments from the same patient could be categorized into distinct subclasses of GBM (10). Several groups propose that treatment failure and recurrence of this tumor type is a consequence of intratumoral heterogeneity (24–26). It is tempting to hypothesize that most changes would occur when comparing the GBM fragments from 1 year apart; yet, our results prove otherwise.

**Figure 4:**
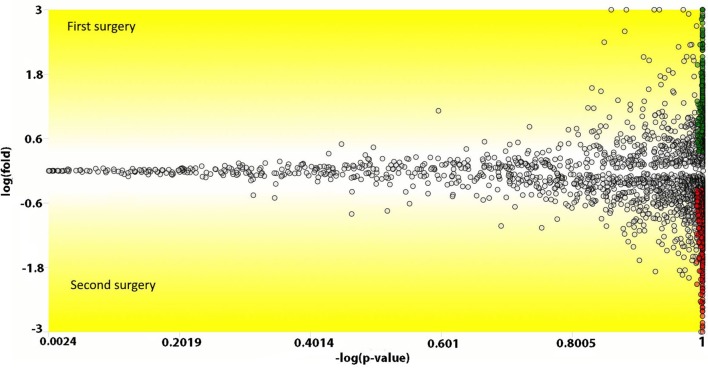
**Volcano plot comparing peptides (the dots) from the profound glioblastoma multiforme (GBM) area derived from the first versus the second surgery**. The *y*-axis represents the Log_e_ fold change and the *x*-axis the −log of the paired *t*-test *p*-value. Peptides (dots) with a positive *y*-value had a higher abundancy in the first surgery; likewise, negative values, are peptides with higher abundancy in the second surgery. Green and red dots represent peptides that achieved a *p*-value lower than 0.01 and an absolute fold change in abundance greater than 1.5.

**Figure 5:**
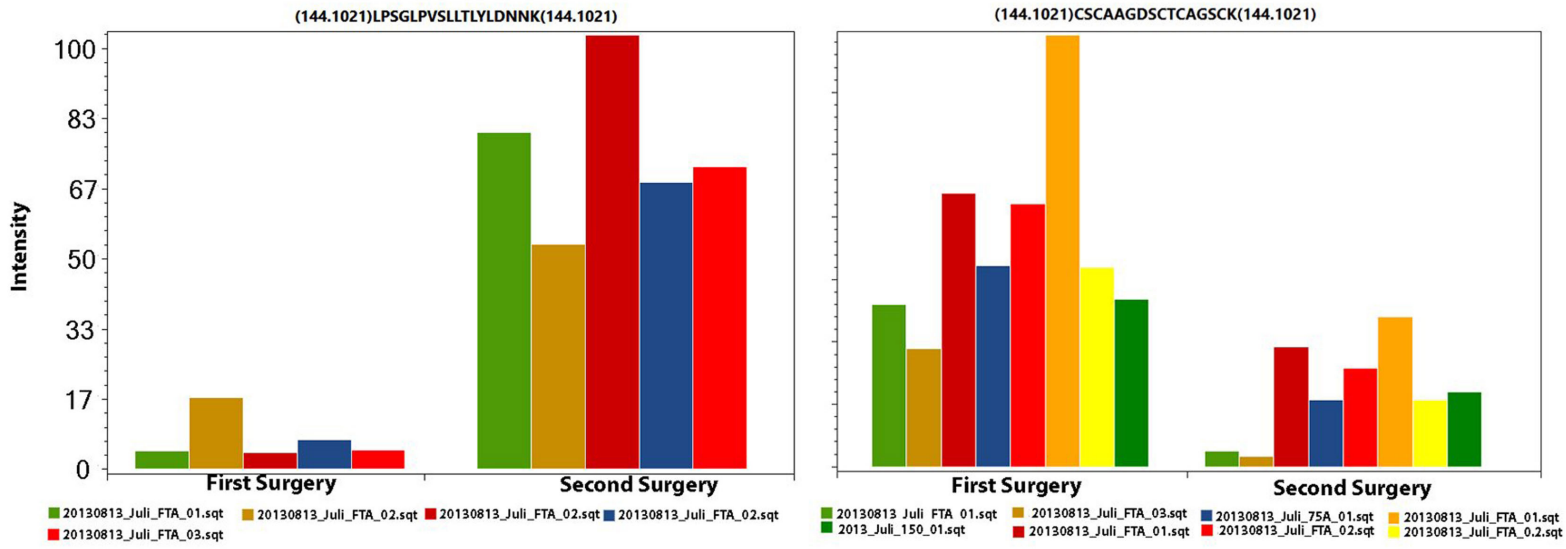
**The iTRAQ normalized signals from peptides mapping to Lumican (left panel) and to Metallothionein (right panel)**. In each plot, each pair of columns with the same color are derived from the same spectrum (markers 114 and 115); this information is necessary for calculating the paired *t*-test. The left and right charts originate from five and eight spectra, respectively.

We used the PatternLab’s isobaric analyzer module to further pursue the differentially abundant proteins between the tissues of the profound regions obtained from different surgeries. We recall that this module employs a peptide-centric approach to assigned paired *t*-test *p*-values to each peptide and then converge to a final *p*-value, at the protein level, through the Stouffer’s method. We applied stringency filters to only consider the signal from unique peptides, proteins with *p*-value < 0.01, and presenting an average absolute peptide fold change greater than 1.5. Figure 4 shows a volcano plot of these results. Table 1 lists selected proteins satisfying these criteria. The complete list of the differentially abundant proteins is addressed in Table S9 in Supplementary Material. Figure 5 demonstrates the normalized iTRAQ signals from peptides mapping to the Metallothionein (P02795) and Lumican (P51884) proteins, upregulated in the first and second surgery, respectively.

**Table 1:** **Differentially abundant proteins obtained when comparing the profound glioblastoma multiforme tissues from the first versus the second surgery**.

ID	Seq	Spec	Fold change	Description
P51884	6	51	−1.666	Lumican
P04271	3	20	−1.456	Protein S100-B
Q562Z6	3	40	−1.371	Actin-like protein (Fragment)
P12109	3	16	−1.338	Collagen alpha-1 (VI) chain
P07339	3	24	−1.295	Cathepsin D
P09382	5	59	−1.287	Galectin-1
P08670	29	592	−1.237	Vimentin
P06703	2	8	−1.052	Protein S100-A6
Q06830	5	36	−0.988	Peroxiredoxin-1
P04792	4	25	−0.865	Heat shock protein beta-1
P30041	2	18	−0.816	Peroxiredoxin-6
P13591	2	8	−0.755	Neural cell adhesion molecule 1
P29966	2	26	−0.751	Myristoylated alanine-rich C-kinase substrate
P50454	4	37	−0.735	Serpin H1
P07237	5	36	−0.727	Protein disulfide-isomerase
P06733	8	92	−0.702	Alpha-enolase
P16152	2	17	−0.696	Carbonyl reductase [NADPH] 1
P30101	3	21	−0.629	Protein disulfide-isomerase A3
P06576	3	23	−0.603	ATP synthase subunit beta, mitochondrial
P09211	3	13	−0.603	Glutathione *S*-transferase P
P30044	2	9	−0.552	Peroxiredoxin-5, mitochondrial
P08758	4	53	−0.401	Annexin A5
Q9H3Z4	2	11	0.123	DnaJ homolog subfamily C member 5
P01024	4	19	0.699	Complement C3
P01023	3	11	0.744	Alpha-2-macroglobulin
P04040	2	7	0.756	Catalase
P02647	2	7	0.932	Apolipoprotein A-I
Q6P5S8	6	65	1.228	IGK@ protein
A8K008	3	35	1.229	cDNA FLJ78387
P26022	2	11	1.244	Pentraxin-related protein PTX3
P02768	4	23	1.321	Serum albumin
P02795	2	23	1.357	Metallothionein-2
Q96K68	2	7	1.37	cDNA FLJ14473 fis, clone MAMMA1001080, highly similar to Homo sapiens SNC73 protein (SNC73) mRNA
P02763	2	15	1.438	Alpha-1-acid glycoprotein 1
P05164	5	30	1.967	Myeloperoxidase
P06702	5	57	2.281	Protein S100-A9

*The ID column lists the Uniprot ID, Seq lists the peptide (sequence) count, Spec lists the spectral count, fold change lists the average Log_e_ fold change of the unique peptides belonging to that protein. All proteins in the Table have a *p*-value <0.01 according to the paired *t*-test for being differentially abundant. A positive fold change indicates the protein to be more abundant in the first surgery*.

Proteins presenting differential abundance between the surgeries held 1 year apart could be linked to recurrence or resistance to anti-cancer agents. We now shortlist several proteins from Table 1 that presented the greatest changes in abundance (*p* < 0.01). S100-A9, myeloperoxidase, catalase and metallothionein-2 were upregulated in the sample from the first surgery and lumican, cathepsin D, galectin-1, and the S100-B protein in the second.

### Proteins Upregulated in the First Surgery

S100 describes proteins from a family that play key roles in multiple stages of tumorigenesis and progression (27). According to Li et al., these proteins, at relatively low concentrations, promote angiogenesis by increasing proliferation, migration, and tube formation of vascular endothelial cells (28). Interestingly, Huang et al. reports S100-A9 to be upregulated in gliomas; however, treatments with NS-398 or aspirin lead to a downregulation of this protein (29). These facts, together with our results, pose the S100A9 as an interesting follow-up marker candidate for this type of tumor.

The myeloperoxidase is a lysosomal enzyme found in neutrophils and monocytes. Its atypical expression can lead to oxidative damage in normal tissues, contributing for the emergence of diseases such as atherosclerosis, Alzheimer, and cancer (30, 31). This protein has been correlated to ovarium (31), lung (32), and prostate cancer (33), to name a few. A recent study suggested that myeloperoxidase activity is essential during the tumoral development; in the later stages; its expression is independent of the progression of cancer cells (34), once again corroborating with our results.

The catalase is an antioxidant enzyme, which play a role in cell defense against oxidative stress (35). In a study with mammary cancer cells, Glorieux et al. observed that higher abundances of this protein decreases the cellular growth and proliferation (36). On the other hand, in a report with lung cancer cells, it was suggesting that catalase may regulate the activity of cathepsin, resulting in a high variation of migration and invasion ability for the cancer development (37). The mechanism of catalase in cancer tumors remains unclear.

The metallothionein-2 was recently reported to be a negative regulator of apoptosis (38). Some studies showed an increased abundance of this protein to be correlated to tumor grade and a proliferative activity in solid tumors, linked to poor survival; therefore, this protein could be a protagonist in carcinogenesis and drug resistance; our results reinforce what was previously demonstrated in some cancer cells, including GBMs (38–40).

### Proteins Upregulated in the Second Surgery

Lumican is a small leucine-rich proteoglycan (SLRPs) that plays roles in cell adhesion and by serving as a regulatory molecule in cellular functions such as cell migration, proliferation, and apoptosis (41, 42). Studies in colorectal, pancreatic, and breast cancer correlated an increased abundance to tumoral growth and metastasis (42–44). Farace et al. noted that the presence of cancer stem cells (CSCs) or tumor-initialing cells, in neuroblastomas and gliomas, promotes the activation of high abundance of SLRPs, creating a cell-microenvironment to survival and possibly favoring cancer recurrence (45).

The cathepsin D is an aspartyl endoproteinase, its higher abundance has been correlated with poor prognosis in breast and glioma cancer patients (46–48). Studies associated its molecular functions with protein catabolism, tissue remodeling, and in multiple tumor progression steps such as proliferation, angiogenesis, and apoptosis (46, 49). According to Berchem et al., this protein stimulates cancer cell proliferation and tumor angiogenesis, independently of its proteolytic activity (49).

Galectin-1 belongs to the carbohydrate-binding proteins family, it is abundantly secreted by almost all types of malignant tumor cells and its expression is tightly linked to the tumor aggressiveness. In particular, this protein plays vital pro-tumorigenic roles within the tumor microenvironment as the suppression of immune responses, promotes the tumor angiogenesis, and others functions related to invasion and metastasis (50, 51). A study with GBM cells revealed that the diffuse gliomas demonstrated higher expression levels compared with pilocytic astrocytoma, which the galectin-1 may modulate the migration and invasion in these cells (52).

The S100-B protein has been used in clinical studies to evaluate the staging malignant melanoma and the treatment success, being considering an independent prognostic factor to predict the survival rate of the patients (53). The increasing S100-B concentrations indicate tumor progression and a worse prognosis for the patient (53); this is coherent with our results, which the patient presents in the second surgery, a high abundance of this protein. The patient deceased 1 year and 1 month after the second surgery.

### Gene Ontology Enrichment Analysis

PatternLab’s Gene Ontology Explore module was employed to statistically pinpoint over-represented gene ontology terms according to the hypergeometric distribution. The completed list of the enriched terms is long, and made available as Table S10 in Supplementary Material. Among some of the most statistically enhanced terms (*p* << 0.01) many are related to the Ras pathway, frequently uncontrolled in gliomas (54). The Ras protein belongs to a familly of small GTPases that is ubiquitously expressed in all cell lineages and organs. This family is responsible for cellular signal transduction bringing to cell growth, differentiation, and survival. The clinically most notable members of the Ras subfamily are H-Ras, K-Ras, and N-Ras, mostly for being involved in many types of cancer (55–57). The enriched terms related to this pathway were: Ras protein signal transduction (GO: 0007265), regulation of Ras protein signal transduction (GO: 0046578) and positive regulation for cell development (GO: 0010720), growth (GO: 0030307), migration (GO: 0030335), and proliferation (GO: 0008284). Among these terms, protein members of the Ras family are frequently found; some examples are: Rab GDP dissociation inhibitor alpha (P31150), Ras-related protein Rap-1A (P62834), Rho GDP-dissociation inhibitor (J3QQX2), Rho GDP-dissociation inhibitor 1 (P52565), Rho GTPase-activating protein 1 (Q07960), Transforming protein RhoA (P61586), Importin subunit beta-1 (Q14974), Rho GDP-dissociation inhibitor 2 (P52566), Ras-related protein Rap-2c (Q9Y3L5), Ras-related protein Rap-2a (P10114). Some other key proteins related to tumors, identified in our dataset, and pertaining to these terms are: Cofilin-1 (P23528), 14-3-3 protein beta/alpha (P31946), Apolipoprotein A-I, E, C-III, Flotillin-1 (P35241) Radixin (P35241, A7YIJ8).

## Final Considerations

Our strategy enabled us to peek behind a GBM’s heterogeneity smokescreen. Nevertheless, our glimpse remains elusive toward probing the real complexity of the cellular networks connecting a GBM’s core to its outer rims. We postulate that spatial comprehension is fundamental for paving the way toward effective treatments and have concentrated efforts in this direction. For example, one of our previous reports explored the proteomic landscape of a gastric cancer total resection (9); here, besides assessing the molecular topology, temporal changes were considered. As far as we know, this is the first work to accomplish such using an isobaric labeling approach. In the midst of so much heterogeneity, so many questions remain. Are the changes detected in the profound region a mechanism of survival? Or a causation from drug resistance? This scenario brings to forefront the discussion on how proteomic experimental design is carried as many reports do not consider spatial proteomics; how much will elevate numbers of “blended” tumors continue to shed light on our knowledge of this disease? We argue there is an entire universe to explore within a single tumor sample and that every tumor is unique. It is time to put into practice and pursue new paradigms devoted toward single-cell proteomics (58), these results could ultimately lead to a better comprehension on the cellular networks that drive tumors and demystify what appears to be a chaotic heterogeneity. Indeed, many say cancer is a loss of cellular control, chaos. Yet, according to José Saramago, a Portuguese writer and Nobel laureate in literature (1988), “chaos is merely order waiting to be deciphered.” Likewise, science argues that heterogeneity, by itself, could be a biomarker as more heterogeneous tumors have a higher chance of containing treatment-resistant subclones (59). So is this heterogeneity really chaos? A loss of order? Or is it a smokescreen camouflaging answers to the very basic questions such as what is the role of cancer in life. Be as it may, if life really emerged from chaos, let the words of Sun Tzu describe its origin: “In this midst of chaos, there is also opportunity.”

## Author Contributions

PA, JF, FN, and GD performed sample preparation and design of experiment. CF, MC, and JS are medical doctors providing contributions related to the medical aspect. PC was responsible for data analysis; other authors that participated in this step were PA, FN, JF, NZ. PC, JF, and PC wrote the manuscript, and all authors reviewed it and made contributions.

## Conflict of Interest Statement

The authors declare that the research was conducted in the absence of any commercial or financial relationships that could be construed as a potential conflict of interest. The reviewers WF and AP and handling Editor declared their shared affiliation, and the handling Editor states that the process nevertheless met the standards of a fair and objective review.

## Supporting Information

The raw mass spectra files, the PatternLab intermediary files, search database, the SEPro identification files and Excel spreadsheets listing the protein identification data, are available for download at http://proteomics.fiocruz.br/supplementaryfiles/oncology2016. PatternLab for proteomics is freely available for download at http://patternlabforproteomics.org
